# Development and validation of a prognostic prediction model for elderly patients with sepsis-associated acute kidney injury based on interpretable machine learning

**DOI:** 10.3389/fmed.2026.1956632

**Published:** 2026-10-16

**Authors:** Nan Zhang, Jian-Zhong Wang, Qi Ma, Jin-Hai Tian, Wen-Jie Zhou

**Affiliations:** 1 Department of Anesthesia and Perioperative Medicine, General Hospital of Ningxia Medical University, Yinchuan, China; 2 Department of Critical Care Medicine, Peking University First Hospital Ningxia Women and Children's Hospital, Yinchuan, China; 3 Department of Critical Care Medicine, Ningxia Hui Autonomous Region Hospital of Integrated Traditional Chinese and Western Medicine, Yinchuan, China; 4 Institute of Medical Sciences, General Hospital of Ningxia Medical University, Yinchuan, China; 5 Department of Critical Care Medicine, General Hospital of Ningxia Medical University, Yinchuan, China

**Keywords:** elderly patients, explainable artificial intelligence, machine learning, prognostic prediction model, sepsis-associated acute kidney injury

## Abstract

**Background:**

Elderly patients with sepsis-associated acute kidney injury (SA-AKI) have high short-term mortality; early identification of high-risk individuals is critical for personalized intervention. Traditional clinical scoring systems relying on linear assumptions and conventional machine learning models often lack interpretability, hindering translation to clinical practice. This study aimed to develop and externally validate an interpretable prognostic model for 28-day in-hospital mortality in elderly SA-AKI using routinely available clinical variables.

**Methods:**

Clinical data of 11,468 elderly SA-AKI patients were retrospectively extracted from the MIMIC-IV database and divided into training and internal test sets at a 7:3 ratio using stratified random sampling. A dual feature selection strategy combining LASSO regression and random forest was applied. Five machine learning models—decision tree, logistic regression, random forest, LightGBM, and XGBoost—were constructed in parallel. Model discrimination and calibration were evaluated by AUC, Brier score, accuracy, sensitivity, specificity, and *F*1 score. External independent validation was performed using a clinical cohort of 257 patients from the General Hospital of Ningxia Medical University. The SHAP method was used for global and individual-level interpretability of the optimal model.

**Results:**

Dual feature selection identified 10 independent predictors: age, weight, red blood cell distribution width, arterial partial pressure of oxygen, blood lactate, prothrombin time, activated partial thromboplastin time, blood urea nitrogen, serum creatinine, and respiratory rate. Among the five models, XGBoost demonstrated the best performance, with training AUC 0.9898 (accuracy 0.9472) and external validation AUC 0.9156 (accuracy 0.8560), indicating strong generalizability. SHAP global importance ranking showed age as the primary driver, followed by serum creatinine and arterial partial pressure of oxygen. Each core feature exhibited a nonlinear association with 28-day mortality risk, with clear model-derived risk inflection points rather than clinical intervention thresholds.

**Conclusion:**

The XGBoost-based interpretable model exhibits excellent discriminative performance and robust external generalizability for predicting 28-day mortality in elderly SA-AKI. The SHAP attribution framework renders model decisions transparent, providing quantitative evidence-based support for early risk stratification and hypothesis generation for individualized organ-protective strategies in the ICU.

## 1 Introduction

Sepsis-associated acute kidney injury (SA-AKI) is one of the most frequent and lethal complications in the intensive care unit (ICU). Its core mechanisms involve systemic inflammatory cascades and haemodynamic disturbances that together mediate renal microvascular endothelial injury, tubular epithelial apoptosis, and mitochondrial dysfunction, further aggravating effective perfusion deficits, promoting metabolic waste accumulation and internal environment imbalance, and ultimately driving multiple organ dysfunction syndrome (1–3). These pathological cascades significantly prolong ICU length of stay and increase short-term mortality, imposing a heavy economic burden (4). Elderly individuals are particularly vulnerable to SA-AKI because of age-related nephron loss, reduced renal blood flow and glomerular filtration reserve, compounded by multiple comorbidities (e.g., hypertension, type 2 diabetes, heart failure) and polypharmacy, which severely impair renal compensatory capacity (5, 6). Epidemiological data indicate that SA-AKI occurs in 15%–20% of ICU patients and accounts for nearly 50% of all acute kidney injury cases (7–9). Short-term mortality in elderly SA-AKI patients is significantly higher than in younger populations, making it a pressing clinical challenge in critical care medicine (10).

Current clinical risk stratification relies mainly on traditional severity scores such as the Sequential Organ Failure Assessment (SOFA) and Acute Physiology and Chronic Health Evaluation III (APACHE III). However, these tools have inherent limitations in mechanistic fitting and individualised warning: they are built on linear additive assumptions, treating each predictor as independently and linearly affecting prognosis, thus failing to capture the nonlinear pathophysiology of SA-AKI involving inflammation-coagulation crosstalk, renal threshold effects, and multi-organ interactions (11, 12). Moreover, the population-level risk stratification they provide does not meet the demands of precision medicine for individualised intervention. In recent years, machine learning algorithms have demonstrated advantages in processing high-dimensional clinical data and identifying nonlinear associations, significantly improving prognostic prediction accuracy (13, 14). Nevertheless, existing machine learning studies face two major methodological bottlenecks. First, some previous models directly include composite scores such as SOFA and APACHE as model input variables, which can cause feature information overlap and weaken the potential to discover novel independent predictors. Second, the internal decision-making logic of ensemble algorithms such as gradient-boosted trees and random forests remains opaque, and their “black-box” nature severely undermines clinician trust and practical model translation (15, 16).

To balance predictive accuracy and clinical interpretability, we introduce the SHAP (SHapley Additive exPlanations) explainable artificial intelligence framework (17). The aim is to build a prognostic model for short-term mortality in elderly SA-AKI that relies solely on routine admission laboratory tests, vital signs, and demographic indicators. The study design proceeded as follows: based on the MIMIC-IV large critical care database, a modelling cohort was constructed according to Sepsis-3.0 and KDIGO AKI diagnostic criteria; LASSO regression and random forest were jointly applied for dual feature selection to avoid redundancy and enhance parsimony; five machine learning models—decision tree, logistic regression, random forest, LightGBM, and XGBoost—were built in parallel and validated through both internal test set and external independent clinical cohort; finally, SHAP was used to quantify each feature's marginal contribution to mortality risk, enabling global variable importance ranking, nonlinear risk inflection point identification for individual features, and visualisation of individual-level risk attribution. Through this approach, we aim to achieve both high predictive performance and decision transparency, providing a quantitative evidence-based tool for early risk stratification and hypothesis generation for individualised organ-protective strategies in elderly SA-AKI patients in the ICU.

## 2 Materials and methods

### 2.1 Study design and data sources

This was a multicentre retrospective cohort study. The modelling dataset was derived from the MIMIC-IV (version 3.0) critical care database, which contains complete electronic health records of emergency and ICU patients admitted to Beth Israel Deaconess Medical Center (2008–2019). Data use was approved by the institutional ethics committee (approval No. 2001 P 001699/14), and the researchers completed human subjects protection training (course No. 59721902) and obtained official access. The external independent validation dataset comprised 257 elderly SA-AKI patients admitted to the Department of Critical Care Medicine, General Hospital of Ningxia Medical University, from October 2023 to November 2025; this retrospective study was approved by the hospital ethics committee (approval No. KYLL 2025 1293) and registered for research.

### 2.2 Study population and screening process

The patient screening flow is shown in Figure 1. Inclusion criteria: (1) age ≥60 years; (2) meeting both Sepsis-3.0 criteria for sepsis (18) and KDIGO criteria for AKI (19, 20); (3) missing rate <30% for all core predictor variables. Exclusion criteria: (1) ICU length of stay <48 h (to exclude selection bias from early death or rapid transfer); (2) age <60 years; (3) history of chronic kidney disease, chronic renal failure, or prior AKI (to avoid confounding from pre-existing chronic renal impairment); (4) a missing rate ≥30% for core clinical variables; (5) initiation of continuous renal replacement therapy (CRRT) within 24 h of admission (to avoid masking the natural course and outcome by early intensive intervention). After applying these criteria, the modelling cohort comprised 11,468 elderly SA-AKI patients, and the external validation cohort included 257 patients. The prediction time point was explicitly defined as the baseline assessment at SA-AKI diagnosis. All candidate predictors were restricted to information available at or before this time point, and treatment information occurring after this time point was not incorporated into model development.

**Figure 1:**
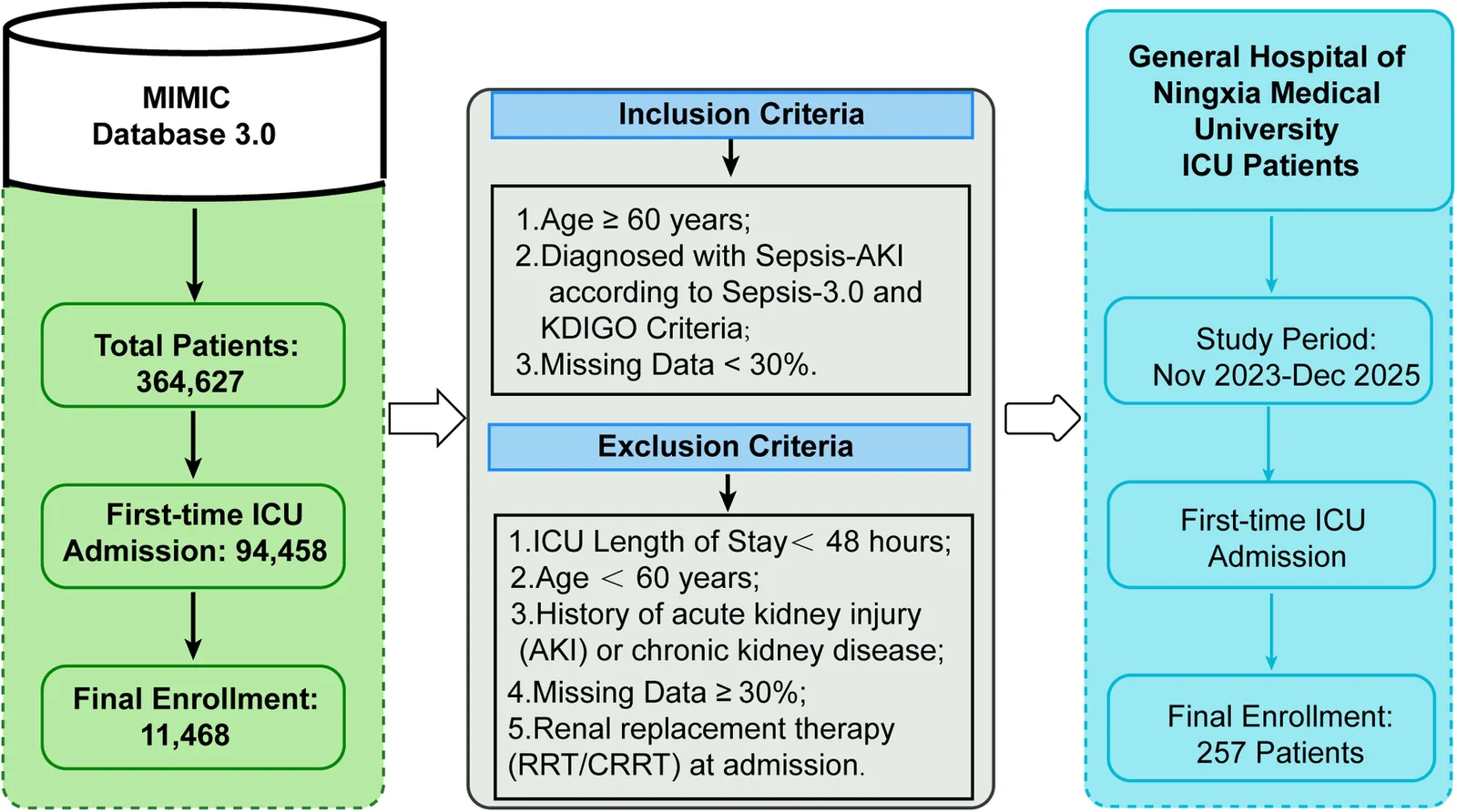
Patient recruitment flowchart for the derivation and external validation cohorts.

### 2.3 Predictor variables: collection and definition

We systematically collected six categories of candidate predictors (detailed in Table 1): (1) demographic indicators: age, weight and gender; (2) baseline comorbidities: diabetes with hypertension, type 2 diabetes alone, diabetes with heart failure and diabetes with myocardial infarction; (3) vital signs: noninvasive systolic, diastolic, mean arterial pressure, respiratory rate, pulse oximetry and body temperature; (4) laboratory tests: complete blood count, electrolytes, glucose, arterial blood gas parameters, coagulation panel and renal function panel; (5) organ function scores: AKI stage, SOFA score, APS III (Acute Physiology Score III) and Glasgow Coma Scale (GCS) score; (6) treatment variables: whether CRRT was administered. CRRT was defined as any CRRT received from ICU admission to SA-AKI diagnosis. Because patients who received CRRT within the first 24 h of admission were excluded, in the final analysed cohort this variable effectively reflects CRRT exposure after the first 24 h and before SA-AKI diagnosis. CRRT after SA-AKI diagnosis was not used as a predictor. Although CRRT was included as a candidate predictor in the initial screening, it did not enter the final model; therefore, the final prediction model does not depend on the CRRT variable. In the preprocessing phase, variables with >30% missingness were first excluded, followed by exclusion of cases with ≥30% missingness in the core variables. Remaining missing values were imputed using the missForest random forest iterative imputation method to maintain data completeness and statistical power (21). Missing-data imputation was performed after the training/test split. The missForest imputation model was developed using the training data only and was then applied to the internal test set; the test set did not contribute to the construction of the imputation model.

**Table 1:** Baseline characteristics of survivors and non-survivors in the overall modelling cohort.

Variables	Statistic	Survival (*N* = 8,550)	Death (*N* = 2,918)	*P*-value
Age	Mean ± SD	77.2 ± 7.9	79.8 ± 8.2	<0.001
Weight	Mean ± SD	82.3 ± 23.5	75.8 ± 20.4	<0.001
Gender	Female	3,738 (43.7%)	1,382 (47.4%)	
	Male	4,812 (56.3%)	1,536 (52.6%)	<0.001
Diaht	No	4,448 (52%)	1,796 (61.5%)	
	Yes	4,102 (48%)	1,122 (38.5%)	<0.001
Diadm2	No	5,520 (64.6%)	1,920 (65.8%)	
	Yes	3,030 (35.4%)	998 (34.2%)	0.227
Diahf	No	5,205 (60.9%)	1,535 (52.6%)	
	Yes	3,345 (39.1%)	1,383 (47.4%)	<0.001
Diami	No	7,885 (92.2%)	2,574 (88.2%)	
	Yes	665 (7.8%)	344 (11.8%)	<0.001
AKI_stage	1	2,101 (24.6%)	318 (10.9%)	<0.001
	2	4,569 (53.4%)	1,159 (39.7%)	<0.001
	3	1,880 (22%)	1,441 (49.4%)	<0.001
CRRT	No	8,207 (96%)	2,580 (88.4%)	<0.001
	Yes	343 (4%)	338 (11.6%)	
Lab RBC	Mean ± SD	3.5 ± 0.6	3.4 ± 0.7	0.001
Lab platelet count	Mean ± SD	196.8 ± 99.8	203.6 ± 115.9	0.002
Lab hemoglobin	Mean ± SD	10.4 ± 1.8	10.1 ± 1.9	<0.001
Lab RDW	Mean ± SD	15.1 ± 2.1	16.2 ± 2.5	<0.001
Lab hematocrit	Mean ± SD	31.5 ± 5.3	31.4 ± 5.8	0.371
Lab sodium	Mean ± SD	138.6 ± 4.7	138.9 ± 5.9	0.002
Lab potassium	Mean ± SD	4.2 ± 0.6	4.3 ± 0.6	0.001
Lab calciumtotal	Mean ± SD	8.3 ± 0.7	8.2 ± 0.8	0.002
Lab chloride	Mean ± SD	104.7 ± 6.0	103.9 ± 7.3	0.001
Lab glucose	Mean ± SD	141.3 ± 52.3	150.3 ± 60.5	0.001
Lab aniongap	Mean ± SD	14.2 ± 3.5	16.1 ± 4.3	0.001
Arterial pH	Mean ± SD	7.4 ± 0.1	7.4 ± 0.1	0.372
Arterial PaCO_2_	Mean ± SD	41.5 ± 8.1	41.5 ± 10.1	0.687
Arterial PaO_2_	Mean ± SD	148.2 ± 78.6	109.0 ± 57.0	0.001
Lab lactate	Mean ± SD	2.0 ± 1.1	2.5 ± 1.9	0.001
Lab totalCO_2_	Mean ± SD	25.3 ± 4.5	24.4 ± 5.7	0.001
Lab PT	Mean ± SD	16.3 ± 7.4	19.1 ± 10.7	0.001
Lab PTT	Mean ± SD	38.3 ± 17.4	43.0 ± 21.8	0.001
Lab INR	Mean ± SD	1.5 ± 0.7	1.8 ± 1.0	0.001
Lab ureanitrogen	Mean ± SD	10.4 ± 7.5	14.4 ± 9.5	0.001
Lab creatinine	Mean ± SD	132.6 ± 114.9	159.1 ± 123.8	0.001
Vitalnbps	Mean ± SD	115.0 ± 16.6	112.6 ± 16.3	0.001
Vitalnbpd	Mean ± SD	61.5 ± 62.2	62.8 ± 72.8	0.397
Vitalnbpm	Mean ± SD	73.7 ± 10.8	73.3 ± 11.1	0.098
Vitalrr	Mean ± SD	19.2 ± 3.7	20.8 ± 4.6	0.001
VitalspO_2_	Mean ± SD	97.1 ± 5.1	96.7 ± 2.6	0.001
Vital temperature	Mean ± SD	36.8 ± 1.6	36.7 ± 2.0	0.212
SOFA	Mean ± SD	5.6 ± 3.0	7.2 ± 3.8	0.001
APS Ⅲ	Mean ± SD	48.1 ± 18.4	62.9 ± 22.7	0.001
GCS	Mean ± SD	13.3 ± 3.0	12.8 ± 3.4	0.001

Diaht, diabetes combined hypertension; Diadm2, type2 diabetes mellitus; Diahf, diabetes combined with heart failure; Diami, diabetes combined with myocardial infarction; AKI_stage, acute kidney injury stage; CRRT, continuous renal replacement therapy; RBC, red blood count; RDW, red cell distribution width; pH, potential of hydrogen; pCO_2_, partial pressure of carbon dioxide; pO_2_, partial pressure of oxygen; PT, prothrombin time; PTT, partial thromboplastin time; INR, international normalized ratio; Vitalnbps, non-invasive systolic blood pressure; Vitalnbpd, non-invasive diastolic blood pressure; Vitalnbpm, non-invasive mean blood pressure; Vitalrr, respiratory rate; VitalspO_2_, pulse oxygen saturation; SOFA, sequential organ failure assessment score; APS Ⅲ, acute physiology score Ⅲ; GCS, Glasgow coma scale. Categorical variables are presented as number (percentage); continuous variables are expressed as mean ± standard deviation. Between-group comparisons were performed using *χ*^2^ test for categorical variables and independent samples *t*-test for continuous variables. *P* < 0.05 was considered statistically significant.

### 2.4 Outcome measure

The primary endpoint was 28-day in-hospital all-cause mortality, defined as death occurring in hospital within 28 days from the date of SA-AKI diagnosis. This endpoint is a well-accepted objective, hard outcome in critical care prognostication, reflecting the extent of organ injury, treatment response, and short-term clinical trajectory. All outcome data were completely extracted from the electronic health records with no loss to follow-up.

### 2.5 Statistical analysis

(1) Dataset split and class balance: The MIMIC-IV original cohort was divided into training and internal test sets at a 7:3 ratio using stratified random sampling. The training set was used for feature selection, model training, and hyperparameter tuning; the internal test set was kept entirely unseen during model development and used only for evaluating internal generalisability. To address class imbalance (survival/death) in the training set, SMOTE (Synthetic Minority Over-sampling Technique) was applied to balance the positive and negative samples to 1:1. SMOTE was applied only to the training set. During cross-validation, synthetic sampling was performed only on the training fold of each fold, while the validation fold retained its original distribution and was not involved in synthetic sampling. The internal test set and external validation set retained their original natural distributions to objectively reflect real-world predictive performance. (2) Dual ensemble feature selection: To select stable and clinically interpretable predictors, we combined LASSO regression (22) and random forest (23) in a dual selection strategy: (i) LASSO regression: 10-fold cross-validation was used to select the *λ*_1_ₛₑ value (largest *λ* within one standard error of the minimum deviance), retaining variables with nonzero coefficients; (ii) random forest: an ensemble of 500 trees was built, with variable importance measured by the mean decrease in Gini impurity, and the top 15 variables were extracted. The intersection of the two sets formed the base feature set. If the intersection contained fewer than 10 variables, we performed normalised weighted scoring based on the absolute LASSO coefficient rank and random forest importance rank, sequentially adding variables by descending score to reach 10 core predictors (this number was pre-specified to control model dimensionality and prevent overfitting). (3) Machine learning model construction and hyperparameter optimisation: Five prediction models were built in parallel: Decision Tree, Logistic Regression, Random Forest, LightGBM and XGBoost. Grid search combined with 5-fold cross-validation was used to systematically tune key hyperparameters (learning rate, tree depth, subsampling ratio, L1/L2 regularisation coefficients). For each model, the optimal classification threshold was determined only in the training set, specifically the SMOTE-balanced training set, by maximizing the Youden index: Youden index = Sensitivity + Specificity − 1. This training-derived threshold was then kept fixed and applied unchanged to both the internal test set and the external validation set when generating binary classifications and confusion matrices. We did not re-optimize or re-estimate the threshold in the internal test set or external validation set. (4) Model performance evaluation: Discriminative ability was assessed by the area under the ROC curve (AUC), along with accuracy, sensitivity, specificity, precision and *F*1 score. AUCs in the external validation set were reported with 95% confidence intervals. Calibration was evaluated using the Brier score (range 0–1; lower indicates better agreement between predicted probabilities and observed outcomes). Calibration and decision curve analyses were also performed. (5) SHAP interpretability analysis: Based on game-theoretic Shapley values, the SHAP additive attribution framework was applied to the optimal XGBoost model for global and individual-level interpretation: (i) global feature importance ranking via SHAP summary plots; (ii) nonlinear associations and model-derived risk inflection points for each core feature via SHAP dependence plots; (iii) individual-level risk attribution via SHAP waterfall plots, thereby visualising and demystifying the model's “black-box” decisions. The model output was on the log-odds scale, and therefore all SHAP values were interpreted on the log-odds scale rather than as changes in predicted probability. (6) Statistical software and significance: All analyses were conducted using R 4.3.3, with packages including missForest, xgboost, randomForest, pROC, caret, SHAPforxgboost, and shapviz. Baseline data were described as counts (percentages) for categorical variables; continuous variables were presented as mean ± SD if normally distributed or median (IQR) if not. Between-group comparisons used two-sided tests, with *P* < 0.05 considered statistically significant.

## 3 Results

### 3.1 Baseline clinical characteristics of the study cohort

A total of 11,468 elderly SA-AKI patients were enrolled, including 8,550 survivors and 2,918 non-survivors, yielding an in-hospital mortality rate of 25.4%. Baseline characteristics of the two groups are shown in Table 1.

Demographics: Non-survivors were significantly older (79.8 ± 8.2 vs. 77.2 ± 7.9 years, *P* < 0.001) and had lower weight (75.8 ± 20.4 vs. 82.3 ± 23.5 kg, *P* < 0.001). The proportion of females was higher in the non-survivor group (47.4% vs. 43.7%, *P* < 0.001).

Comorbidities: Non-survivors had a significantly lower rate of diabetes with hypertension (38.5% vs. 48%, *P* < 0.001), whereas diabetes with heart failure (47.4% vs. 39.1%, *P* < 0.001), and diabetes with myocardial infarction (11.8% vs. 7.8%, *P* < 0.001), whereas the prevalence of type 2 diabetes alone did not differ between groups (34.2% vs. 35.4%, *P* = 0.227).

AKI severity and treatment: AKI stage distribution differed significantly (*P* < 0.001). AKI stage 2 predominated in survivors (53.4%), whereas nearly half of non-survivors (49.4%) had stage 3, far higher than the 22% in survivors. Correspondingly, CRRT utilisation was significantly higher in non-survivors (11.6% vs. 4%, *P* < 0.001).

Laboratory findings: Non-survivors had significantly higher red blood cell distribution width (16.2 ± 2.5 vs. 15.1 ± 2.1, *P* < 0.001), lactate (2.5 ± 1.9 vs. 2.0 ± 1.1, *P* < 0.001), prothrombin time (19.1 ± 10.7 vs. 16.3 ± 7.4, *P* < 0.001), international normalised ratio (1.8 ± 1.0 vs. 1.5 ± 0.7, *P* < 0.001), blood urea nitrogen (14.4 ± 9.5 vs. 10.4 ± 7.5, *P* < 0.001), and creatinine (159.1 ± 123.8 vs. 132.6 ± 114.9, *P* < 0.001); whereas haemoglobin (10.1 ± 1.9 vs. 10.4 ± 1.8, *P* < 0.001), arterial partial pressure of oxygen (109.0 ± 57.0 vs. 148.2 ± 78.6, *P* < 0.001), and total carbon dioxide (24.4 ± 5.7 vs. 25.3 ± 4.5, *P* < 0.001) were significantly lower. Haematocrit, blood pH, and partial pressure of carbon dioxide did not differ significantly between groups (all *P* > 0.05).

Vital signs and severity scores: Non-survivors had higher respiratory rate (20.8 ± 4.6 vs. 19.2 ± 3.7 breaths/min, *P* < 0.001) and lower systolic blood pressure (112.6 ± 16.3 vs. 115.0 ± 16.6 mmHg, *P* < 0.001). Regarding severity, non-survivors had higher SOFA (7.2 ± 3.8 vs. 5.6 ± 3.0, *P* < 0.001) and APS III (Acute Physiology Score III) scores (62.9 ± 22.7 vs. 48.1 ± 18.4, *P* < 0.001), and lower GCS scores (12.8 ± 3.4 vs. 13.3 ± 3.0, *P* < 0.001), indicating more severe organ failure and worse overall physiological status at admission.

In summary, univariate analysis identified advanced age, low weight, cardiovascular comorbidities, advanced AKI stage, coagulation abnormalities, more severe renal impairment, elevated lactate, and higher severity scores as factors associated with in-hospital mortality, which were then included in subsequent multivariable analysis for model construction.

### 3.2 Results of combined LASSO and random forest feature selection

LASSO regularisation retained 10 variables with nonzero coefficients, which were included in the candidate set; simultaneously, the top 15 variables from random forest importance ranking completely overlapped with the LASSO-selected variables, requiring no additional weighted supplementation. The final 10 core predictors covered seven clinical dimensions: demographics, renal function, oxygenation, tissue perfusion, coagulation, inflammation, and respiratory vital signs (Table 2): age, weight, red blood cell distribution width (RDW), arterial partial pressure of oxygen (PaO_2_), blood lactate, prothrombin time (PT), activated partial thromboplastin time (APTT), blood urea nitrogen (BUN), serum creatinine (Scr), and respiratory rate (RR).

**Table 2:** Results of feature selection.

Feature name	LASSO selection	LASSO coefficient	Random forest rank	Random forest importance
Age	Yes	0.0246	13	127.485
Weight	Yes	−0.0134	3	173.23
RDW	Yes	0.1006	4	166.078
PaO_2_	Yes	−0.0042	2	203.789
Lactic acid	Yes	0.0554	9	133.681
PT	Yes	0.0051	11	131.376
APTT	Yes	0.0046	14	126.08
BUN	Yes	0.0074	5	153.052
Scr	Yes	−0.1486	15	121.261
RR	Yes	0.0323	6	150.447

RDW, red cell distribution width; PaO_2_, partial pressure of oxygen; PT, prothrombin time; APTT, activated partial thromboplastin time; BUN, blood urea nitrogen; Scr, serum creatinine; RR, respiratory rate.

LASSO coefficients indicated positive associations with mortality for age, RDW, lactate, PT, APTT, BUN, and RR (coefficients >0), and negative associations for weight, PaO_2_, and Scr (coefficients <0). Random forest importance ranking (descending) was: PaO_2_, weight, RDW, BUN, RR, lactate, PT, age, APTT, Scr, suggesting that oxygenation, weight, and RDW were the top contributing laboratory/demographic features.

### 3.3 Comparison of predictive performance among five machine learning models

Discrimination, calibration, and overall classification performance were evaluated in the training set, internal test set, and external independent validation set; all metrics are summarised in Table 3.

**Table 3:** Results of machine learning models.

Model name	Dataset	AUC	Brier score	Accuracy	Sensitivity	Specificity	Precision	Recall	*F*1-score
Decision tree	Training set	0.7913	0.1827	0.7459	0.7574	0.7343	0.7403	0.7574	0.7488
Decision tree	Test set	0.6849	0.2215	0.6552	0.5554	0.6893	0.3788	0.5554	0.4504
Decision tree	External validation	0.7448	0.1861	0.7471	0.6761	0.7742	0.5333	0.6761	0.5963
LightGBM	Training set	0.8884	0.1466	0.8018	0.7938	0.8097	0.8066	0.7938	0.8002
LightGBM	Test set	0.7407	0.1827	0.6735	0.6846	0.6698	0.4142	0.6846	0.5162
LightGBM	External validation	0.8053	0.1669	0.7743	0.6761	0.8118	0.5783	0.6761	0.6234
Logistic regression	Training set	0.7490	0.2030	0.6855	0.7475	0.6234	0.6650	0.7475	0.7038
Logistic regression	Test set	0.7452	0.2038	0.6930	0.6469	0.7088	0.4311	0.6469	0.5174
Logistic regression	External validation	0.7667	0.2001	0.6420	0.8169	0.5753	0.4234	0.8169	0.5577
Random forest	Training set	0.9437	0.1047	0.8688	0.9006	0.8371	0.8468	0.9006	0.8729
Random forest	Test set	0.7287	0.2155	0.6985	0.5600	0.7458	0.4291	0.5600	0.4859
Random forest	External validation	0.8849	0.1509	0.8054	0.7887	0.8118	0.6154	0.7887	0.6914
XGBoost	Training set	0.9898	0.0626	0.9472	0.9419	0.9525	0.9520	0.9419	0.9469
XGBoost	Test set	0.7283	0.1770	0.7291	0.4411	0.8273	0.4656	0.4411	0.4531
XGBoost	External validation	0.9156	0.1102	0.8560	0.7324	0.9032	0.7429	0.7324	0.7376

Training set performance: Ensemble learning models (XGBoost, random forest, LightGBM) substantially outperformed traditional logistic regression and the single decision tree. XGBoost achieved the highest AUC (0.9898), lowest Brier score (0.0626, indicating best calibration), and the highest accuracy (0.9472), sensitivity (0.9419), specificity (0.9525), and F1 score (0.9469). Random forest ranked second (AUC = 0.9437, Brier = 0.1047), followed by LightGBM (AUC = 0.8884). The traditional models underperformed, with AUCs of 0.7913 for decision tree and 0.7490 for logistic regression.

Internal test set performance: All models showed a marked performance decline, indicating substantial model optimism. AUC ordering was: logistic regression (0.7452) > LightGBM (0.7407) > random forest (0.7287) > XGBoost (0.7283) > decision tree (0.6849). XGBoost had the highest specificity (0.8273) but lower sensitivity (0.4411), suggesting better identification of low-risk survivors but increased missed detection of high-risk deaths; LightGBM showed more balanced sensitivity (0.6846) and specificity (0.6698). Overall, gradient-boosting models exhibited moderate internal generalisability, necessitating external validation to confirm real-world performance.

External independent validation set performance: In the single-centre external cohort, XGBoost demonstrated superior generalisability over other algorithms, with the best overall performance: AUC = 0.9156, accuracy = 0.8560, specificity = 0.9032, *F*1 score = 0.7376, and Brier score = 0.1102, indicating stable probability calibration. The external cohort included 257 patients, among whom 70 deaths occurred (28-day in-hospital mortality 27.2%). The external validation AUCs with 95% confidence intervals are presented in Table 4, and the baseline characteristics of the external cohort are summarized in Table 5. Random forest ranked second (AUC = 0.8849); logistic regression and decision tree showed markedly lower discrimination, with AUCs of 0.7667 and 0.7448, respectively. Considering both high training set discrimination and robust external generalisation, XGBoost was identified as the optimal model for predicting 28-day in-hospital mortality in elderly SA-AKI patients. Corresponding ROC curves, decision curves, and calibration curves are shown in Figure 2 (A–C: training set; D–F: internal test set; G–I: external validation set).

**Table 4:** The external validation AUCs with 95% cis.

Model	Dataset	AUC	95% CI
Decision Tree	External validation	0.7448	0.6737–0.8158
LightGBM	External validation	0.8053	0.7467–0.8640
Logistic Regression	External validation	0.7667	0.7053–0.8281
Random Forest	External validation	0.8849	0.8411–0.9288
XGBoost	External validation	0.9156	0.8767–0.9544

**Table 5:** Baseline characteristics of survivors and non-survivors in the external cohort.

Variables	Survinal (*N* = 187)	Death (*N* = 70)	*P*-value
Age	77.0 ± 8.3	81.4 ± 8.8	0.001
Weight (kg)	83.3 ± 22.4	69.0 ± 16.1	0.001
Gender			
Female	75 (40.1%)	36 (51.4%)	
Male	112 (59.9%)	34 (48.6%)	0.104
History of hypertension			
No	108 (57.8%)	41 (58.6%)	
Yes	79 (42.2%)	29 (41.4%)	0.906
History of type 2 diabetes			
No	115 (61.5%)	43 (61.4%)	
Yes	72 (38.5%)	27 (38.6%)	0.992
History of heart failure			
No	124 (66.3%)	36 (51.4%)	
Yes	63 (33.7%)	34 (48.6%)	0.029
History of myocardial infarction			
No	168 (89.8%)	63 (90%)	
Yes	19 (10.2%)	7 (10%)	0.970
AKI_stage (KDIGO)			
Stage 1	53 (28.3%)	11 (15.7%)	
Stage 2	97 (51.9%)	33 (47.1%)	0.203
Stage 3	37 (19.8%)	26 (37.1%)	0.004
CRRT			
No	175 (93.6%)	65 (92.9%)	
Yes	12 (6.4%)	5 (7.1%)	0.835
Lab RBC (×10^12^/L)	3.5 ± 0.7	3.4 ± 0.8	0.125
Lab platelet count (×10⁹/L)	204.8 ± 93.9	201.6 ± 113.2	0.820
Lab hemoglobin (g/dL)	10.4 ± 1.9	9.8 ± 2.1	0.025
Lab RDW (%)	15.1 ± 2.4	16.3 ± 2.9	0.002
Lab hematocrit (%)	31.8 ± 5.6	30.5 ± 6.5	0.119
Lab sodium (mmol/L)	138.6 ± 5.3	139.5 ± 6.7	0.314
Lab potassium (mmol/L)	4.3 ± 0.6	4.3 ± 0.7	0.989
Lab calciumtotal (mg/dL)	8.2 ± 0.7	8.3 ± 0.8	0.238
Lab chloride (mmol/L)	104.3 ± 5.9	104.8 ± 7.4	0.576
Lab glucose (mmol/L)	14.4 ± 4.7	16.1 ± 6.7	0.033
Lab aniongap (mmol/L)	14.2 ± 3.5	16.3 ± 3.9	0.001
Arterial pH	7.4 ± 0.1	7.4 ± 0.1	0.383
Arterial PaCO_2_ (mmHg)	42.5 ± 9.2	39.0 ± 7.5	0.006
Arterial PaO_2_ (mmHg)	147.7 ± 78.8	108.9 ± 58.8	0.001
Lab lactate (mmol/L)	2.0 ± 1.0	2.5 ± 1.6	0.014
Total CO_2_ (mmol/L)	25.7 ± 5.3	23.2 ± 4.4	0.001
Lab PT (s)	15.9 ± 7.2	17.6 ± 6.7	0.098
Lab PTT (s)	37.9 ± 17.9	45.5 ± 24.2	0.010
Lab INR	1.5 ± 0.7	1.6 ± 0.7	0.078
Lab ureanitrogen (mmol/L)	10.9 ± 9.6	17.5 ± 12.7	0.001
Lab creatinine (*μ*mol/L)	141.4 ± 123.8	194.5 ± 185.6	0.005
Vitalnbps (mmHg)	115.8 ± 17.5	113.3 ± 18.8	0.312
Vitalnbpd (mmHg)	60.1 ± 9.7	59.7 ± 10.2	0.785
Vitalnbpm (t/m)	73.7 ± 10.8	74.4 ± 20.7	0.707
Vitalrr (t/m)	18.9 ± 3.8	21.5 ± 5.0	0.001
VitalspO_2_ (%)	96.9 ± 1.9	97.0 ± 2.1	0.798
Vital temperature (℃)	36.8 ± 0.5	36.8 ± 0.5	0.851
SOFA	5.5 ± 3.2	7.1 ± 3.9	0.002
APS Ⅲ	49.4 ± 17.3	63.6 ± 22.3	0.001
GCS	13.2 ± 3.3	12.9 ± 3.5	0.576

AKI_stage, acute kidney injury stage; CRRT, continuous renal replacement therapy; RBC, red blood count; RDW, red cell distribution width; pH, potential of hydrogen; pCO_2_, partial pressure of carbon dioxide; pO_2_, partial pressure of oxygen; PT, prothrombin time; PTT, partial thromboplastin time; INR, international normalized ratio; Vitalnbps, non-invasive systolic blood pressure; Vitalnbpd, non-invasive diastolic blood pressure; Vitalnbpm, non-invasive mean blood pressure; Vitalrr, respiratory rate; VitalspO_2_, pulse oxygen saturation; SOFA, sequential organ failure assessment score; APS Ⅲ, acute physiology score Ⅲ; GCS, Glasgow coma scale. Categorical variables are presented as number (percentage); continuous variables are expressed as mean ± standard deviation. Between-group comparisons were performed using *χ*^2^ test for categorical variables and independent samples *t*-test for continuous variables. *P* < 0.05 was considered statistically significant.

**Figure 2:**
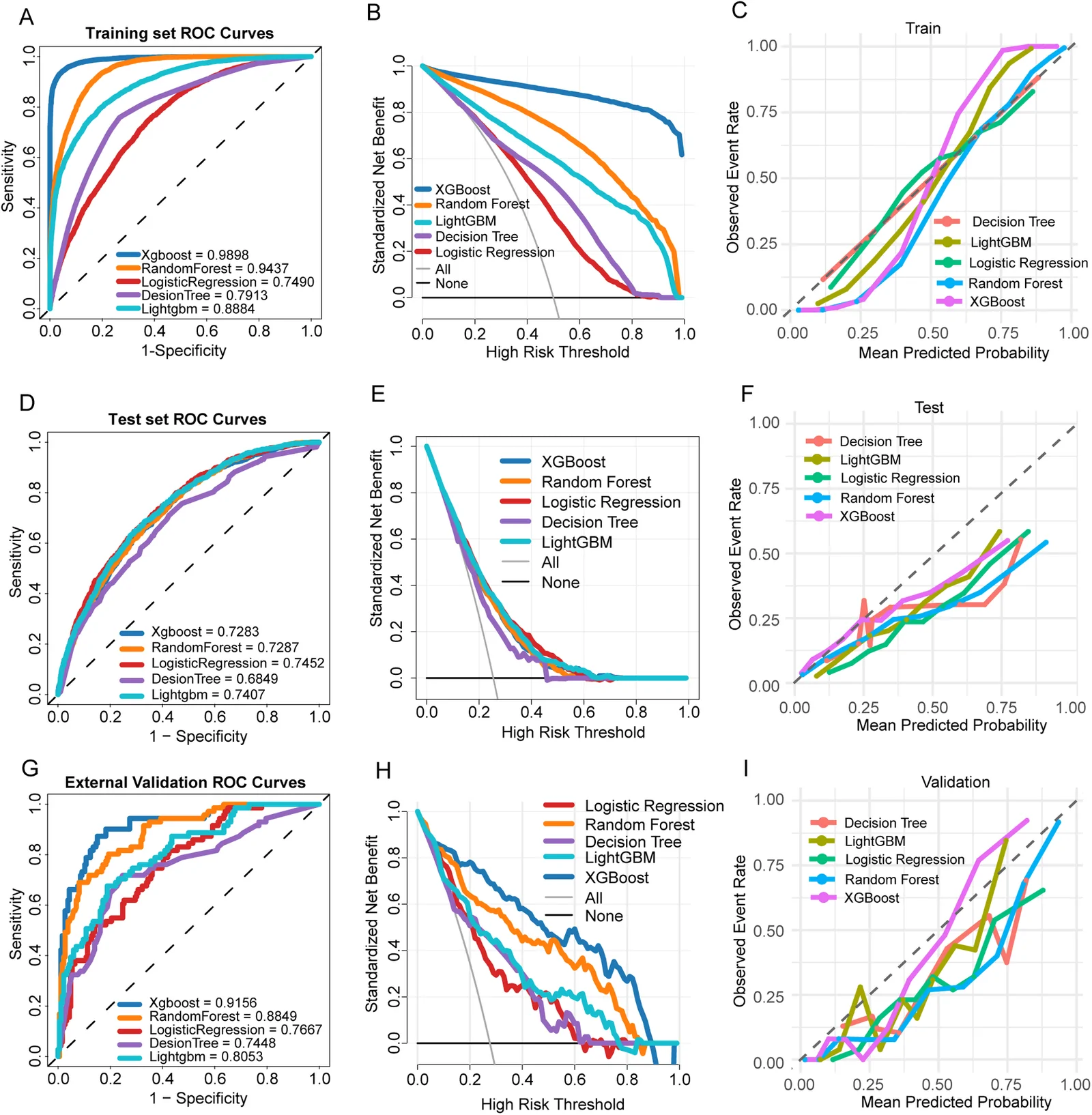
Comparison of predictive performance of five machine learning models on the training set, internal test set, and external independent validation set. **(A–C)** Training set; **(D–F)** Internal test set; **(G–I)** External independent validation set. **(A,D,G)** ROC curves of each model for evaluating discriminative ability; **(B,E,H)** decision curve analysis, quantifying the clinical net benefit of models at different risk thresholds, with the black horizontal line representing the strategy of no intervention for all, and the gray horizontal line representing the strategy of intervention for all; **(C,F,I)** calibration curves, with the dashed diagonal line representing the ideal calibration state. XGBoost, extreme gradient boosting; LightGBM, light gradient boosting machine.

### 3.4 SHAP interpretability analysis of the optimal XGBoost model

To overcome the “black-box” limitation, we applied the SHAP algorithm to the optimal XGBoost model for global and individual-level interpretation, clarifying the role of each clinical variable in mortality prediction. Because the model output was on the log-odds scale, all SHAP values below are reported on the log-odds scale. Visualisations are presented in Figures 3, 4.

**Figure 3:**
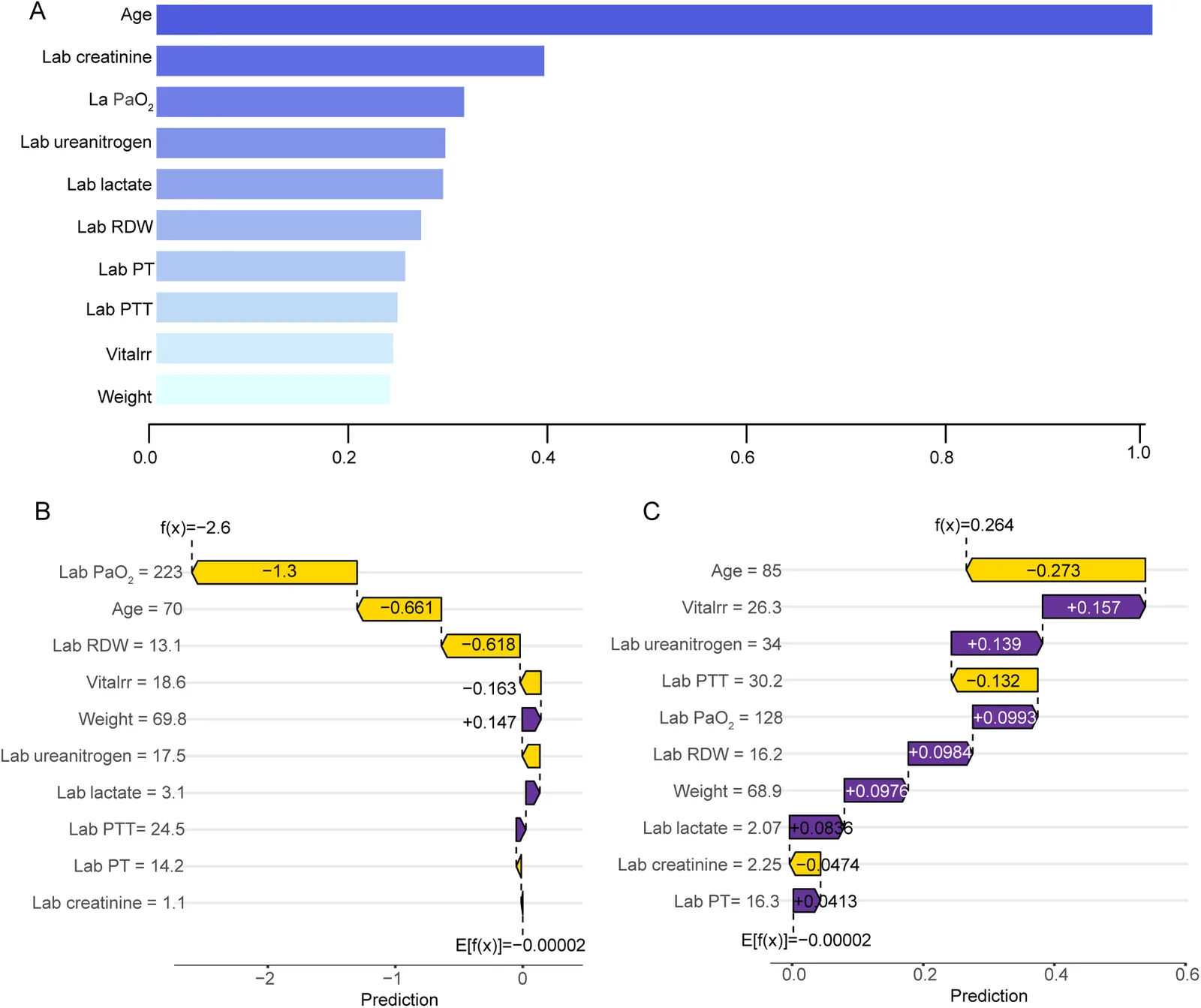
SHAP global importance and individualized waterfall plots of the XGBoost model. **(A)** Bar chart of feature importance ranking based on mean absolute SHAP values; **(B)** SHAP waterfall plot for a patient with low mortality risk; **(C)** SHAP waterfall plot for a patient with high mortality risk. E[f(x)] represents the model baseline output value (mean predicted risk across the entire sample), and f(x) represents the final predicted log-odds of mortality risk for that patient.

**Figure 4:**
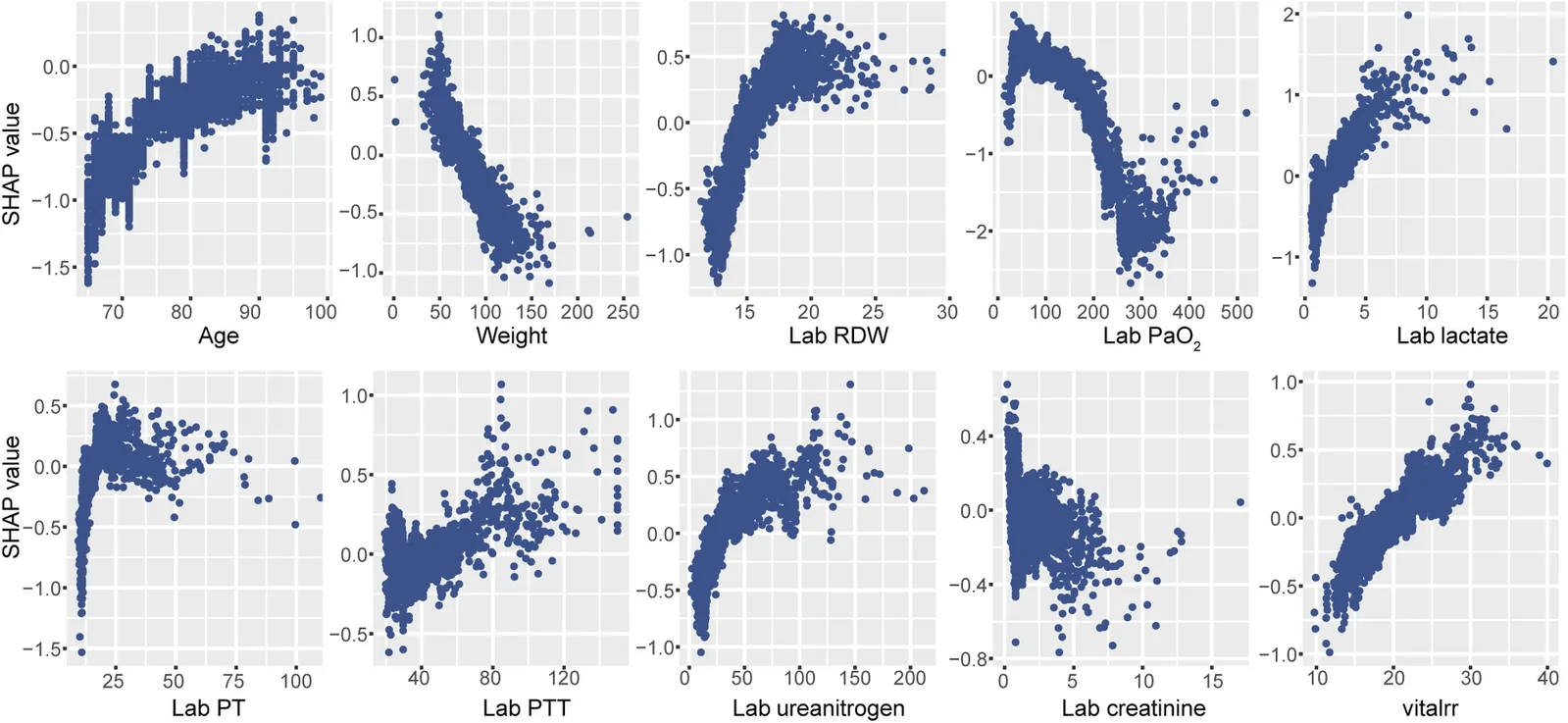
SHAP dependence plots of core predictive variables. The *X*-axis represents the measured values of the corresponding clinical indicators, and the *Y*-axis represents SHAP values; positive SHAP values indicate an increased predicted mortality risk, while negative SHAP values indicate a decreased predicted mortality risk. The curves in the figures reflect the non-linear trends between each indicator and mortality risk.

#### 3.4.1 Global SHAP feature importance

Based on mean absolute SHAP values across all samples, the normalised importance ranking showed that age was the top predictor, with a substantially higher contribution than other variables, followed by renal and oxygenation indicators: serum creatinine and arterial partial pressure of oxygen; blood urea nitrogen, lactate, RDW, respiratory rate, PT, APTT, and weight followed in descending order. These results indicate that age-related physiological decline, acute renal injury, and tissue hypoperfusion/hypoxia are the three core dimensions determining short-term mortality risk in SA-AKI (Figure 3A).

#### 3.4.2 Global SHAP feature importance

4.2 Individual-level SHAP waterfall plots: Typical high-risk and low-risk cases were selected to illustrate the positive/negative marginal contributions of each variable to the model output, with the baseline model output (log-odds) set as the average log-odds of the entire cohort (Figures 3B,C).

1. High-risk case: On top of the baseline prediction, respiratory rate (26.3 breaths/min, SHAP = +0.157) and BUN (34 mmol/L, SHAP = +0.139) were the main positive drivers; although age (85 years) slightly decreased the risk (SHAP = −0.273), multiple factors including elevated lactate, hypoxia, and low weight collectively contributed positive risk, resulting in an overall high-risk classification.
2. Low-risk case: PaO_2_ of 223 mmHg was the strongest protective factor (SHAP = −1.3), markedly reducing the model output; younger age (70 years, SHAP = −0.661) and RDW of 13.1% (SHAP = −0.618) synergistically exerted protective effects, pushing the predicted log-odds well below the baseline and leading to a low-risk survival classification.

Individual-level SHAP analysis confirms that the model does not rely on a single threshold but integrates multidimensional clinical information (demographics, renal function, oxygenation, coagulation, respiration) to make a comprehensive assessment. Even in the presence of advanced age, adequate oxygenation, stable renal function, and normal lactate levels can substantially offset the mortality risk, and the model's reasoning aligns closely with clinical pathophysiology.

### 3.5 SHAP dependence plots: nonlinear associations and threshold effects

SHAP single-feature dependence plots visualise the nonlinear relationship between each core predictor and in-hospital mortality, and identify several model-derived risk inflection points. These values are risk-associated ranges derived from the model and should not be interpreted as clinical intervention thresholds or therapeutic targets (Figure 4).

1. Age: SHAP values increased monotonically with age, with a population-level approximate risk inflection point at 78 years. Below 78 years, SHAP values tended to remain negative, indicating a generally protective association; above 78 years, SHAP values tended to turn positive and rose with age. However, this should not be interpreted as a strict individual-level cutoff. The age-related SHAP value for an individual patient is also influenced by the remaining features and feature interactions; therefore, an 85-year-old patient may still show a negative age-related SHAP contribution in a specific case.
2. Blood lactate: Lactate showed a stepwise positive nonlinear association with mortality. Below 2 mmol/L, SHAP values stayed low, indicating manageable risk; once lactate exceeded approximately 2 mmol/L, SHAP values rose sharply, with increasing lactate levels paralleling higher mortality risk. This value is a model-derived risk inflection point and does not by itself provide a quantitative reference for early fluid resuscitation or organ support.
3. Respiratory rate: Increasing respiratory rate drove SHAP values upward, with risk amplification beyond the normal physiological range, suggesting that tachypnoea is a risk-associated early warning sign and can be used for bedside dynamic monitoring.
4. Arterial partial pressure of oxygen: A model-derived risk inflection point around 200 mmHg was identified. When PaO_2_ < 200 mmHg, SHAP values were markedly elevated, with hypoxaemia substantially increasing short-term mortality risk; above 200 mmHg, SHAP values declined rapidly. This value should not be regarded as a therapeutic oxygen target without prospective validation.

## 4 Discussion

In this study, using the MIMIC-IV large critical care database combined with a real-world single-centre external cohort, we integrated a dual feature selection strategy (LASSO + random forest) and successfully developed an XGBoost-based interpretable prognostic model for 28-day in-hospital mortality in elderly SA-AKI patients. Baseline analysis showed that non-survivors were older, had lower weight, higher proportions of diabetes with heart failure/myocardial infarction, more advanced AKI stage, higher CRRT utilisation, and exhibited impaired oxygenation, lactate accumulation, coagulation disturbance, inflammatory activation, and worse multi-organ function scores—all differences statistically significant and highly consistent with the pathophysiology of septic kidney injury (24, 25). Systemic inflammation and haemodynamic instability compromise renal perfusion, and on top of age-related renal reserve decline, further induce tubular damage, metabolic waste accumulation, and internal milieu derangement, ultimately amplifying multi-organ failure and short-term mortality-providing solid pathophysiological support for the 10 core predictors selected.

The dual-algorithm selection yielded 10 routine clinical indicators—age, weight, RDW, PaO_2_, lactate, PT, APTT, BUN, Scr, and respiratory rate—all rapidly obtainable, non-invasive, low-cost parameters available at ICU admission, without needing complex specialised tests or composite scores, thus offering strong potential for clinical implementation. Multi-model comparison confirmed that ensemble learning algorithms (XGBoost, random forest, LightGBM) consistently outperformed traditional logistic regression and single decision tree, aligning with previous findings that ensemble methods excel in handling high-dimensional nonlinear clinical data (26). Among them, XGBoost demonstrated both high training set discrimination (AUC = 0.9898) and robust external generalisation (AUC = 0.9156), with Brier scores in the ideal range. The marked difference between the training and internal test AUC indicates substantial model optimism. Several factors may explain this difference: the training set was SMOTE-balanced while the internal test set retained the original natural class distribution; hyperparameters were tuned on the training set, which may introduce model optimism; and age, sex, disease severity, comorbidity burden, AKI stage, and treatment patterns may differ across the training, internal test, and external cohorts. We also confirmed that the training/internal test split was performed at the patient level to avoid leakage from repeated ICU admissions. In addition, the higher AUC in the external validation cohort than in the internal test set should be interpreted cautiously and not taken as evidence that the model generalises better externally than internally. The external cohort was a single-centre, more homogeneous cohort with a smaller sample size and limited number of outcome events; therefore, its AUC estimate may be less stable and subject to spectrum bias. We have added baseline comparisons among the training set, internal test set, and external cohort, including age, sex, SOFA/APS III, AKI stage, and outcome, with standardised mean differences, and have revised the Discussion so that the external AUC is presented as supportive rather than directly superior to the internal test AUC. SHAP interpretability analysis further revealed the underlying clinical logic: age was the leading risk driver, with a population-level approximate risk inflection point at 78 years; serum creatinine and PaO_2_ followed, representing renal injury severity and tissue oxygenation status; lactate, respiratory rate, RDW and other indicators showed nonlinear associations with model-derived risk inflection points. Elevated lactate (>approximately 2 mmol/L), hypoxaemia (<200 mmHg), and increased respiratory rate stepwise raised mortality risk, whereas adequate oxygenation, stable renal function, and younger age substantially offset adverse effects. Individual waterfall plots confirmed that the model does not rely on a single determinant but integrates multi-dimensional information, fully reflecting the multi-organ interactive nature of elderly SA-AKI and addressing the core “black-box” limitation of conventional machine learning (27, 28).

Previous SA-AKI prognostic studies fall into two main categories: traditional linear scoring systems and conventional machine learning models. First, classic severity scores such as SOFA and APACHE III are built on linear additive assumptions, treating predictors independently and failing to capture nonlinear interactions among inflammation, coagulation, renal, and respiratory systems (29–31). Our baseline data showed no significant group differences in pCO_2_, diastolic pressure, or body temperature, yet traditional scores forcibly include many non-predictive variables, causing redundancy and reduced stratification precision. Multiple cohort studies have reported AUCs for traditional scores in elderly SA-AKI short-term mortality mostly ranging 0.65–0.78 (32, 33), markedly inferior to our externally validated XGBoost model (AUC = 0.9156), and they only provide population-level stratification without individualised risk quantification. Second, existing machine learning models have two clear shortcomings. (1) Some previous models directly include composite scores like SOFA and APACHE as inputs, causing feature overlap and masking the independent predictive value of basic parameters such as blood counts, blood gases, and renal function, thus hindering the discovery of simple novel biomarkers (16, 34). Our study completely avoided composite scores as inputs, using only raw clinical variables, and identified 10 bedside-available indicators better suited for resource-limited ICUs. (2) Most existing machine learning studies report only discrimination metrics like AUC without interpretability analysis, leaving clinicians unable to understand decision logic and thereby hindering translation into intervention (16). Although some studies have introduced SHAP, they typically only present global importance rankings without exploring nonlinear risk inflection points or providing quantitative risk-associated reference values (35–37). Our study, via SHAP dependence plots, identified model-derived risk inflection points for age, lactate, PaO_2_, and respiratory rate, which may inform future hypothesis-driven studies of fluid resuscitation, oxygen therapy, and organ support, but should not be regarded as direct clinical intervention thresholds.

At the subgroup level, most prior AKI prediction models have included ICU patients of all ages without specifically focusing on the elderly high-risk group. The elderly have physiological renal decline and multiple comorbidities, with pathophysiological features significantly heterogeneous from younger adults, and general models may introduce predictive bias (5). Our study strictly restricted inclusion to age ≥60 years, specifically targeting this high-risk subgroup, thus offering greater clinical relevance. Moreover, many similar studies rely solely on internal validation within a single database, lacking independent real-world external validation, raising doubts about generalisability (38). Our study combined the large MIMIC-IV (US) database with a domestic single-centre elderly cohort for both internal and external validation, ensuring population diversity and local clinical applicability, thus providing a higher level of evidence.

Our study establishes a “LASSO + random forest” dual-ensemble feature selection framework that balances linear variable associations and nonlinear interactions, ensuring stability and predictive gain, and offers a standardised pipeline for high-dimensional critical care data. We also present a complete research paradigm of “machine learning modelling + internal/external dual-cohort validation + multi-level SHAP interpretability”, providing a reproducible methodological reference for similar prognostic model development in critical illness. SHAP nonlinear risk inflection point analysis quantitatively confirms that elderly SA-AKI prognosis is collectively modulated by age, renal function, tissue oxygenation, perfusion, and inflammation/coagulation pathways, identifies model-derived risk inflection points (78 years, lactate approximately 2 mmol/L, PaO_2_ 200 mmHg), and from a data-driven perspective supports the “age–oxygenation–renal function” triple-axis injury mechanism, providing clinical epidemiological evidence for future basic mechanistic studies. The study also confirms the superiority of XGBoost gradient boosting in handling heterogeneous elderly critical care data, accurately fitting nonlinear inter-organ feedback relationships compared with linear or single-tree models, and clearly establishes the theoretical advantage of ensemble learning in geriatric critical care prognostication.

By relying on admission routine tests and vital signs, our model enables risk assessment without waiting for complex organ function scores, allowing early identification of elderly SA-AKI patients at high risk of 28-day mortality soon after diagnosis, supporting timely consideration of intensified organ protection, fluid resuscitation, and oxygen therapy in future prospective studies—achieving “early identification, early intervention” precision critical care. SHAP individual waterfall plots can visually display each patient's risk drivers, distinguishing dominant injury pathways (hypoxia, renal injury, hyperlactataemia, advanced age, etc.) and helping clinicians generate hypotheses for individualised treatment plans, moving beyond the “one-size-fits-all” stratification of traditional scores.

Nevertheless, this study has several limitations. First, both the modelling and external validation cohorts were retrospectively collected from electronic health records, which inevitably involve missing data, recording bias, and selection bias; we could not obtain long-term home medications, pre-hospital care, nutritional status, or other potential confounders not documented in the electronic records, which may slightly affect effect estimates and predictive precision. Second, the external validation cohort comprised only 257 elderly SA-AKI patients from a single centre, with limited sample size, limited number of outcome events, and regional population; future multi-centre, larger-scale ICU cohorts are needed to further validate cross-centre and cross-region generalisability. Third, we only used 28-day in-hospital all-cause mortality as the sole outcome, without follow-up for 3-month, 6-month, or 1-year renal recovery, long-term survival, or new-onset chronic kidney disease, so the model's value for long-term prognosis remains unassessed. Fourth, we only performed model development, validation, and interpretability analysis without a prospective cohort study, so we cannot confirm that risk-stratified interventions based on this model can effectively reduce mortality; clinical benefit still requires prospective trials to demonstrate. Fifth, we used only routine clinical laboratory indicators and did not include novel kidney-specific biomarkers such as neutrophil gelatinase-associated lipocalin (NGAL) or kidney injury molecule-1 (KIM-1) (39, 40); future integration of such biomarkers may further improve discrimination. Sixth, this study is limited to offline data analysis without developing a web-based, mobile, or EHR-embedded prediction tool, so real-time bedside automated risk prediction is not yet feasible, presenting a barrier to clinical translation. Seventh, CRRT was defined from ICU admission to SA-AKI diagnosis, and because patients receiving CRRT within the first 24 h were excluded, the CRRT variable reflects a restricted time window; although CRRT did not enter the final model, this definition should be considered when interpreting the results. Eighth, the prediction time point was SA-AKI diagnosis, and all predictors were restricted to information available at or before this time point; treatment information after SA-AKI diagnosis was not incorporated into model development, which limits the model's applicability to later treatment-related decision-making.

Future research should proceed in the following directions. First, multi-centre large-sample prospective studies should be conducted to expand external validation cohorts and adjust for population heterogeneity; randomised controlled trials should compare the effects of individualised intervention based on our XGBoost model vs. conventional SOFA-guided therapy on short- and long-term outcomes in elderly SA-AKI, to clarify the clinical value of the model. Second, incorporate long-term outcomes such as renal function deterioration, 1-year all-cause mortality, and progression to chronic kidney disease to build a composite model covering both short-term mortality and long-term renal prognosis; incorporate novel biomarkers (NGAL, KIM-1, inflammatory cytokines) (41–43) together with imaging or ultrasound renal perfusion parameters to enrich the feature set and further enhance predictive accuracy. Third, based on the optimal XGBoost model, derive a simplified scoring rule and develop a web calculator, EHR plugin, or mobile mini-program that automatically outputs mortality risk probability and SHAP-based individualised interpretation after inputting the 10 routine indicators, streamlining clinical workflow and promoting routine application across ICUs at all levels. Fourth, using the model-derived risk inflection points identified (lactate, PaO_2_, age, respiratory rate), conduct stratified intervention trials to explore optimal target values for different risk drivers and develop standardised clinical pathways for individualised organ protection in elderly SA-AKI. Extend the model framework to other AKI subgroups such as young/middle-aged SA-AKI, postoperative AKI, and cardiorenal syndrome-associated AKI, to compare core predictor differences and clarify mechanistic heterogeneity across aetiologies; simultaneously, with explainable machine learning, build prognostic systems for other critical illnesses such as multi-organ failure and septic shock. Finally, this study only used single baseline measurements at admission for static prediction; future work should incorporate dynamic vital signs and laboratory time-series data at 6, 12, and 24 h after admission to construct time-series dynamic prediction models that update mortality risk in real time, enabling continuous monitoring and early warning.

In summary, this study developed an XGBoost model based on interpretable machine learning that accurately predicts 28-day in-hospital mortality in elderly SA-AKI using only routinely available clinical variables, with internal and external validation confirming excellent discrimination and generalisability. SHAP analysis clarified the nonlinear prognostic effects and model-derived risk inflection points of core features, overcoming the black-box limitation of traditional machine learning. Compared with conventional severity scores and existing similar models, our study offers greater population specificity, simpler indicators, and more comprehensive interpretability, with substantial theoretical methodological innovation and clinical stratification value. Despite limitations including retrospective design and single-centre external validation, this study provides a reliable quantitative tool for early risk warning in elderly SA-AKI. Through future multi-centre prospective validation, lightweight tool development, and dynamic time-series optimisation, this model can be further translated into critical care practice to improve outcomes for elderly patients with sepsis-associated acute kidney injury.

## Data Availability

The original contributions presented in the study are included in the article/Supplementary Material, further inquiries can be directed to the corresponding authors.
